# Unit Cells in Space or Spaces for Unit Cells

**DOI:** 10.1063/4.0000896

**Published:** 2025-10-27

**Authors:** Lawrence C Andrews, Herbert J Bernstein

**Affiliations:** 1 Ronin Institute for Independent Scholarship, Kirkland, WA, USA; 2 Fresh Pond Research Research Institute, Cambridge, MA, USA

## Abstract

Crystallographers think about unit cells as 3 lengths and 3 angles. In other words, as 6 numbers,often in a row. But in order to talk about the relationships between unit cells, we need to know where they are in the space of unit cells, whatever that is. Ideally, we would like to be able to measure the "distances" between 2 or more unit cells, so we would like to have a metric space. The lengths and angles can be treated as a 6- dimensional space, but the they are not commensurate, so there is not a 'nice" measure. Other 6 or 7 dimensional spaces are needed.

